# Targeting hypoxia-inducible factor-1 alpha suppresses *Helicobacter pylori*-induced gastric injury via attenuation of both *cag*-mediated microbial virulence and proinflammatory host responses

**DOI:** 10.1080/19490976.2023.2263936

**Published:** 2023-10-13

**Authors:** Jennifer M. Noto, M. Blanca Piazuelo, Judith Romero-Gallo, Alberto G. Delgado, Giovanni Suarez, Konstantina Akritidou, Miguel Girod Hoffman, Juan Carlos Roa, Cormac T. Taylor, Richard M. Peek

**Affiliations:** a Department of Medicine, Vanderbilt University Medical Center, Nashville, TN, USA; b Department of Biology, Davidson College, Davidson, NC, USA; c School of Medicine, University of Puerto Rico, San Juan, USA; d Department of Pathology, School of Medicine, Center for Cancer Prevention and Control (CECAN), Pontificia Universidad Catolica de Chile, Santiago, Chile; e School of Medicine, Systems Biology Ireland and The Conway Institute, University College Dublin, Dublin, Ireland; f Department of Pathology, Microbiology, and Immunology, Vanderbilt University Medical Center, Nashville, TN, USA

**Keywords:** *Helicobacter pylori*, gastric inflammation, gastric cancer, hypoxia-inducible factor-1 alpha (HIF-1α), dimethyloxalylglycine (DMOG), prolyl hydroxylase (PHD)

## Abstract

*Helicobacter pylori*-induced inflammation is the strongest known risk factor for gastric adenocarcinoma. Hypoxia-inducible factor-1 (HIF-1α) is a key transcriptional regulator of immunity and carcinogenesis. To examine the role of this mediator within the context of *H. pylori*-induced injury, we first demonstrated that HIF-1α levels were significantly increased in parallel with the severity of gastric lesions in humans. In interventional studies targeting HIF-1α, *H. pylori*-infected mice were treated ± dimethyloxalylglycine (DMOG), a prolyl hydroxylase inhibitor that stabilizes HIF-1α. *H. pylori* significantly increased proinflammatory chemokines/cytokines and inflammation in vehicle-treated mice; however, this was significantly attenuated in DMOG-treated mice. DMOG treatment also significantly decreased function of the *H. pylori* type IV secretion system (T4SS) *in vivo* and significantly reduced T4SS-mediated NF-κB activation and IL-8 induction *in vitro*. These results suggest that prolyl hydroxylase inhibition protects against *H. pylori*-mediated pathologic responses, and is mediated, in part, via attenuation of *H. pylori cag*-mediated virulence and suppression of host proinflammatory responses.

## Introduction

*Helicobacter pylori*-induced inflammation and injury is the strongest known risk factor for gastric adenocarcinoma, the fourth leading cause of cancer-related mortality worldwide and which accounts for > 800,000 deaths annually.^1^ Heightened risk for *H. pylori*-mediated pathologic outcomes is orchestrated by complex interactions between *H. pylori* virulence determinants, host immune responses, and the exposome. One *H. pylori* strain-specific virulence determinant that augments disease risk is the *cag* pathogenicity island, and strains harboring this constituent induce more severe gastric injury. The *cag* island encodes a type IV secretion system (T4SS), which translocates the effector protein CagA into host gastric epithelial cells. Intracellular CagA can undergo tyrosine-phosphorylation or remain unphosphorylated; in either form, CagA aberrantly activates numerous signaling pathways which can induce proinflammatory responses.^2–4^ However, only a subset of persons infected by *cag*-positive strains ever develop cancer,^5^ underscoring the importance of defining precise interactions that increase gastric cancer risk.

Environmental conditions also accentuate the risk for *H. pylori*-mediated inflammation and carcinogenesis. Our laboratory previously demonstrated that iron deficiency enhances the ability of *H. pylori* to induce gastric carcinogenesis in rodent models through augmentation of *H. pylori* virulence and alterations in host responses that drive inflammation.^6,7^ Another environmental condition that contributes to *H. pylori*-induced gastric carcinogenesis is oxygen availability, and populations residing at high altitudes are at heightened risk for gastric cancer.^8^ Both oxygen and iron regulate intracellular levels of hypoxia-inducible factors.^9^ Under normoxia, oxygen-dependent prolyl hydroxylases (PHD) are active within the cell and can hydroxylate HIF-1α leading to ubiquitin-mediated proteasomal degradation. Under hypoxic conditions, hydroxylation is inhibited due to inactivation of PHD, and HIF-1α is stabilized and functions as a central mediator of cellular adaptation to hypoxic conditions. Several other factors can induce HIF-1α via regulating reactive oxygen species (ROS) or kinases, and gastric epithelial ROS, whether endogenous or induced by *H. pylori*, enhance HIF-1α expression in gastric mucosa under normoxic conditions.^10^ Importantly, HIFs have emerged as major transcriptional regulators of immunity^11^ and are involved in cancer progression.^12,13^ Thus, we sought to investigate the role of HIF-1α in the development of gastric inflammation and injury within the context of *H. pylori* infection. To address this, we utilized *in vivo*, *ex vivo*, and *in vitro* models of HIF-1α stabilization via DMOG (dimethyloxalylglycine), a cell-permeable inhibitor of PHD, and demonstrate that DMOG is protective in *H. pylori*-induced injury and inhibits the *cag* type IV secretion system-mediated virulence potential of *H. pylori* and corresponding host proinflammatory immune responses.

## Results

### HIF-1α expression is associated with more advanced disease in humans

**Figure 1.**
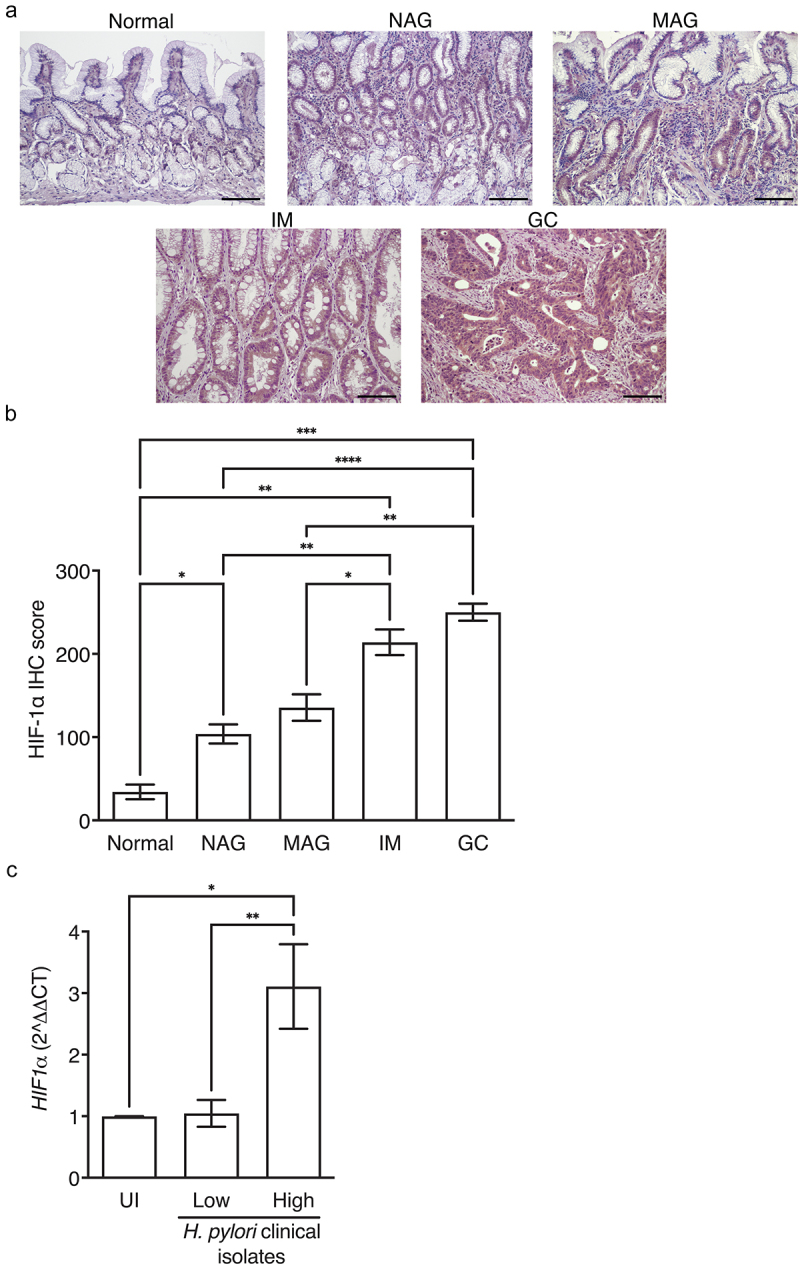
HIF-1*α* is associated with more advanced disease in humans. HIF-1α IHC was quantified in patients with normal gastric mucosa (*N* = 6), non-atrophic gastritis (NAG, *N* = 8), multifocal atrophic gastritis (MAG, *N* = 10), intestinal metaplasia (IM, *N* = 13), and gastric cancer (GC, *N* = 10) (a-b). Representative IHC images are shown at 200X and scale bars represent 100 µm (a). AGS cells were co-cultured with *H. pylori* clinical isolates from low-risk (*N* = 9) or high-risk (*N* = 9) patients and *HIF-1α* expression was assessed by qRT-PCR (c). ANOVAs with Sidak multiple comparisons test (b-c) were used for statistical analyses and standard error of the mean is shown. ****, *P* < .0001; ***, *P* < .001; **, *P* < .01; *, *P* < .05.

HIFs have been shown to play an important role in the progression to cancer. Thus, to address this in a human population at heightened risk for inflammation-driven gastric cancer, we assessed HIF-1α expression levels in gastric tissue from patients with normal gastric mucosa, non-atrophic gastritis, multifocal atrophic gastritis, intestinal metaplasia, or gastric cancer (Supplementary Table S1, (Figure 1a-b)). HIF-1α expression levels paralleled the severity of gastric disease, with the highest levels among patients with gastric cancer (Figure 1a-b).

We next assessed the ability of *H. pylori* clinical strains isolated from patients from low versus high gastric cancer risk regions in Colombia to induce *HIF-1α*. The majority of patients from the low gastric cancer risk region (Pacific coastal town of Tumaco, Colombia) had a histologic diagnosis of non-atrophic gastritis, while the majority of patients from the high gastric cancer risk region (Andean mountain town of Túquerres, Colombia) had more advanced histologic diagnoses of intestinal metaplasia (Supplementary Table 2). Despite the high prevalence of *H. pylori* infection in each region (>90%), patients from the high-risk mountain region harbor a 25-fold increase in cancer rate compared to the low-risk coastal region.^14,15^ All *H. pylori* strains isolated from the low-risk and high-risk patients were urease-, catalase-, and oxidase-positive, and the majority of *H. pylori* strains isolated from the low-risk and high-risk patients were *cagA*^*+*^, *vacA* s1m1, *sabA*^*+*^, and *babA*^*+*^ (Supplementary Table 2). However, the MLST classifications differed between strains isolated from the low-risk versus high-risk patients,^16^ whereby *H. pylori* strains isolated from low-risk patients were hspWAfrica types, while *H. pylori* strains isolated from high-risk patients were hpEurope types (Supplementary Table 2). *H. pylori* clinical strains were co-cultured with gastric epithelial cells and *HIF-1α* expression was assessed by qRT-PCR. *H. pylori* strains isolated from high-risk patients induced significantly higher levels of *HIF-1α*, compared to strains isolated from low-risk patients (Figure 1c), indicating that *H. pylori* strains infecting high-risk mountainous populations may adapt to hypoxia, which could alter *H. pylori* virulence.

### *DMOG treatment paradoxically reduces* H. pylori-*induced injury* in vivo

We next conducted interventional studies targeting HIF-1α using a C57BL/6 mouse model of *H. pylori*-induced inflammation and injury. To first assess the expression of HIF-1α in this model, immunohistochemistry (IHC) was performed on gastric tissue sections from uninfected and *H. pylori*-infected C57BL/6 mice. HIF-1α was quantified in both the gastric epithelium and gastric inflammatory infiltrates. Although there were no significant differences in HIF-1α expression in epithelial cells following infection, *H. pylori* significantly increased levels of HIF-1α in foci of inflammation (Supplementary Figure S1A-C). To then investigate the functional role of HIF-1α in *H. pylori*-induced inflammation and injury, C57BL/6 mice were treated with or without DMOG, a cell-permeable inhibitor of PHD that stabilizes HIF-1α and then challenged with or without *H. pylori*. To first assess the direct effects of DMOG *in vivo*, we quantified levels of HIF-1α as well as carbonic anhydrase IX (CA9), a direct downstream target of PHD, by IHC. DMOG treatment significantly increased levels of HIF-1α in inflammatory infiltrates of *H. pylori*-infected mice (Supplementary Figure S1D) and significantly increased levels of CA9 in the gastric corpus (Supplementary Figure S1E), indicating that DMOG directly leads to HIF-1α stabilization and activation of HIF-1α downstream targets.

**Figure 2.**
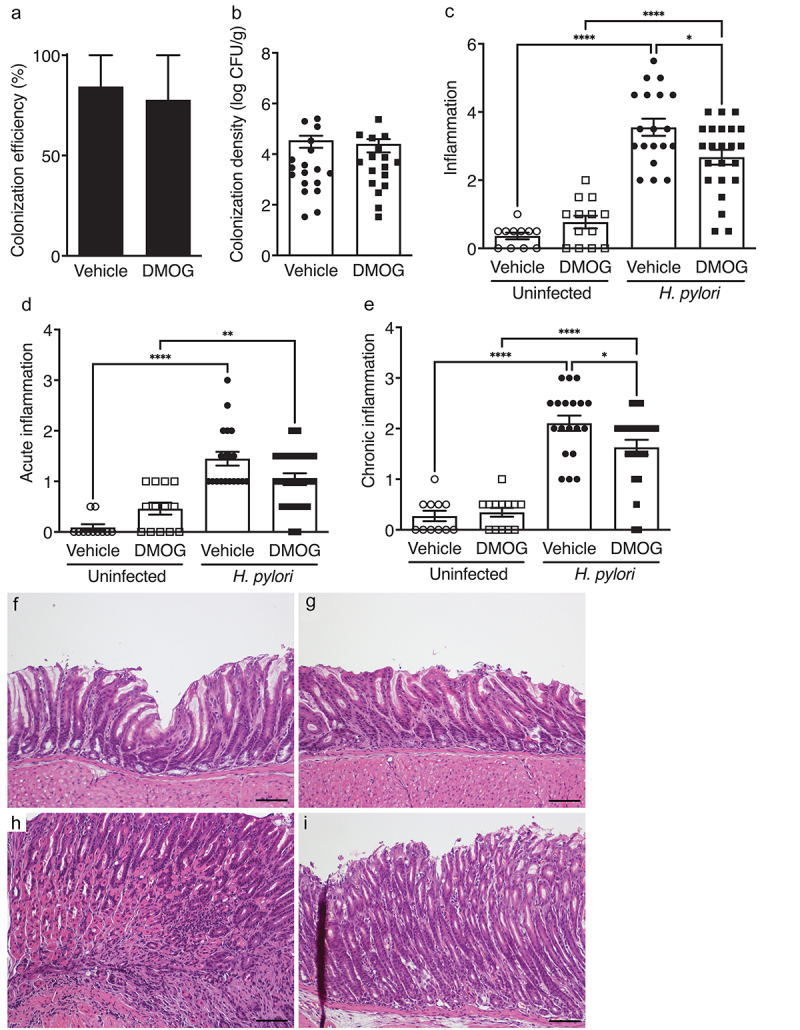
DMOG reduces *H. pylori*-induced inflammation *in vivo*. mice were treated with vehicle (*N* = 30) or DMOG (*N* = 36) and then challenged with or without *H. pylori*. Colonization efficiency (a) and colonization density (b) are shown. Total gastric inflammation (c), acute inflammation (d), and chronic inflammation (e) were quantified and representative H&E images from uninfected mice treated with vehicle (f) or DMOG (g) and *H. pylori*-infected mice treated with vehicle (h) or DMOG (i) are shown at 200X and scale bars represent 100 µm. Open symbols: uninfected mice; closed symbols: *H. pylori*-infected mice. Circles: vehicle-treated; squares: DMOG-treated. Unpaired parametric t-test (b) and one-way ordinary ANOVAs with Sidak multiple comparison test (c-e) were used for statistical analyses and standard error of the mean is shown. ****, *P* < .0001; **, *P* < .01; *, *P* < .05.

Having established the effectiveness of DMOG treatment, *H. pylori* colonization was assessed. DMOG treatment had no effect on *H. pylori* colonization efficiency (Figure 2a) or colonization density (Figure 2b) compared to vehicle-treated controls. We hypothesized that stabilization of HIF-1α would exacerbate *H. pylori*-induced disease *in vivo*; however, DMOG treatment reciprocally reduced *H. pylori*-induced inflammation (Figure 2c-i). When acute and chronic inflammation were independently assessed, DMOG treatment reduced acute inflammation, but this was not statistically significant; however, DMOG significantly reduced levels of chronic inflammation (Figure 2d-e). Collectively, these findings demonstrate that treatment with DMOG is protective in an *in vivo* model of *H. pylori*-induced inflammation and injury.

### *DMOG treatment reduces proinflammatory immune responses and M1 macrophage polarization markers* in vivo

**Figure 3.**
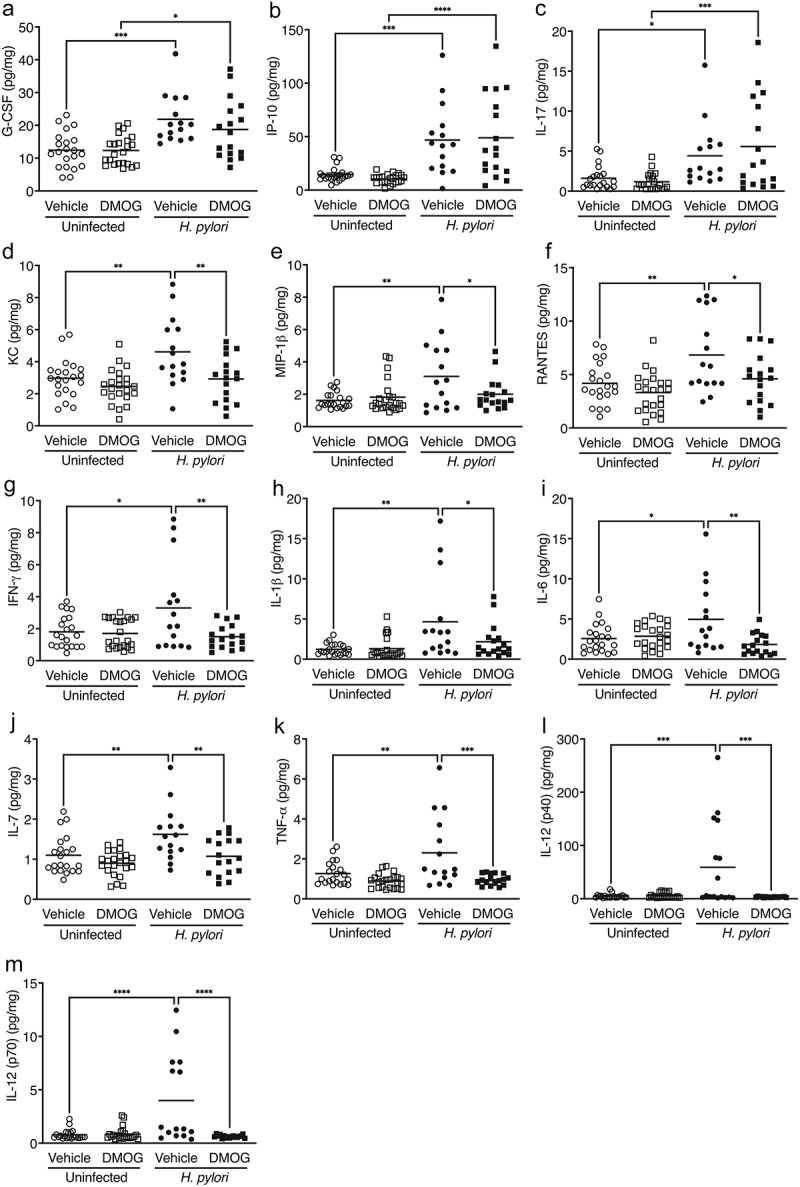
DMOG treatment reduces proinflammatory immune responses and M1 macrophage polarization markers *in vivo*. Chemokines and cytokines were analyzed in gastric tissue from mice treated with vehicle or DMOG and then challenged with or without *H. pylori*. Levels of G-CSF (a), IP-10 (b), and IL-17 (c) were increased with *H. pylori* infection. *H. pylori*-induced chemokines KC (d), MIP-1β (e), and RANTES (f) as well as proinflammatory cytokines INF-γ (g), IL-1β (h), IL-6 (i), IL-7 (j), TNF-α (k), IL-12 (p40) (l), and IL-12 (p70) (m) were significantly attenuated by DMOG treatment. Open symbols: uninfected mice; closed symbols: *H. pylori*-infected mice. Circles: vehicle-treated; squares: DMOG-treated. ANOVAs with Sidak multiple comparisons test were used for statistical analyses. ****, *P* < .0001; ***, *P* < .001; **, *P* < .01; *, *P* < .05.

To define mechanisms by which DMOG paradoxically elicits a protective effect in *H. pylori*-induced injury, we next assessed chemokine and cytokine expression in gastric tissue from uninfected and *H. pylori*-infected mice treated with vehicle or DMOG (Figure 3 and Supplementary Figure S2). *H. pylori* infection induced significant increases in the levels of several proinflammatory chemokines and cytokines, and a subset of these inflammatory mediators were significantly attenuated among infected mice treated with DMOG. *H. pylori* infection, regardless of treatment group, resulted in significant increases in gastric mucosal levels of G-CSF, IP-10, and IL-17 (Figure 3a–c), compared to uninfected controls. *H. pylori* infection of vehicle-treated mice also resulted in significant increases in levels of the chemokines KC, MIP-1β, and RANTES (Figure 3d–f) and the proinflammatory cytokines IFN-γ, IL-1β, IL-6, IL-7, TNF-α, and IL-12 (Figure 3g–m), when compared to uninfected, vehicle-treated mice. However, these chemokine and cytokine proinflammatory responses were significantly attenuated in DMOG-treated mice infected with *H. pylori* (Figure 3d–m).

To further investigate the mechanisms underpinning this phenotype, we next assessed M1 macrophage polarization markers within the context of *H. pylori* infection and DMOG treatment *in vivo*. It has been previously demonstrated that DMOG alters macrophage polarization via reductions in levels of the proinflammatory cytokines IL-1β, IL-6, and TNF-α which drive M1 macrophage polarization.^17–19^ In this model of *H. pylori*-induced gastric injury, in addition to reductions in levels of the previously identified M1 macrophage markers IL-1β, IL-6, and TNF-α (Figures 3h–i, and 3k), DMOG also significantly reduced the M1 macrophage markers IFN-γ, IL-7, IL-12(p40), and IL-12(p70) (Figures 3j and 3l-m). Collectively, these data demonstrate that DMOG treatment significantly reduces proinflammatory immune responses induced by *H. pylori*, and this is likely mediated through reduced M1 macrophage polarization.

Since HIF-1α is an important regulator of immunity and is highly expressed in immune cells, we next employed a novel and enriched primary *ex vivo* model system using cells isolated from a mouse model that is prone to gastric carcinogenesis, INS-GAS mice. To first validate the expression pattern of HIF-1α seen in the C57BL/6 model, IHC was performed on gastric tissue sections from uninfected and *H. pylori*-infected INS-GAS mice and HIF-1α was quantified. As observed in our C57BL/6 mouse model, infection with *H. pylori* significantly increased levels of HIF-1α in foci of inflammation (Supplementary Figure S3A-C). Having validated HIF-1α expression patterns in INS-GAS mice, gastric organoids were generated from these mice and seeded in the upper chamber of a transwell system to form 2D monolayers, while autologous bone marrow-derived macrophages and splenocytes treated with anti-CD3 and anti-CD28, to induce T cell activation, were placed in the lower chamber. Epithelial gastric organoid monolayers were then infected with *H. pylori* and chemokine and cytokine expression was quantified from inflammatory macrophage/T cell lysates. Consistent with our *in vivo* findings (Figure 3), *H. pylori* significantly increased the proinflammatory chemokines *Kc* and *Rantes* (Supplementary Figure S3D-E) as well as proinflammatory cytokines *Il1β*, *Il17*, and *Tnfα* (Supplementary Figure S3F-H). Further, *H. pylori* also significant increased levels of *Il9* and *Il22* (Supplementary Figure S3I-J), cytokines that are regulated by HIF-1α and which contributes to T cell differentiation and immunity.^20–22^ Collectively, these data validate our *in vivo* findings in another mouse model, but also reveal the important contribution of immune cell components in HIF-1α-mediated chemokine and cytokine induction.

### *DMOG treatment attenuates* H. pylori cag *T4SS function and the subsequent induction of downstream proinflammatory responses*

**Figure 4.**
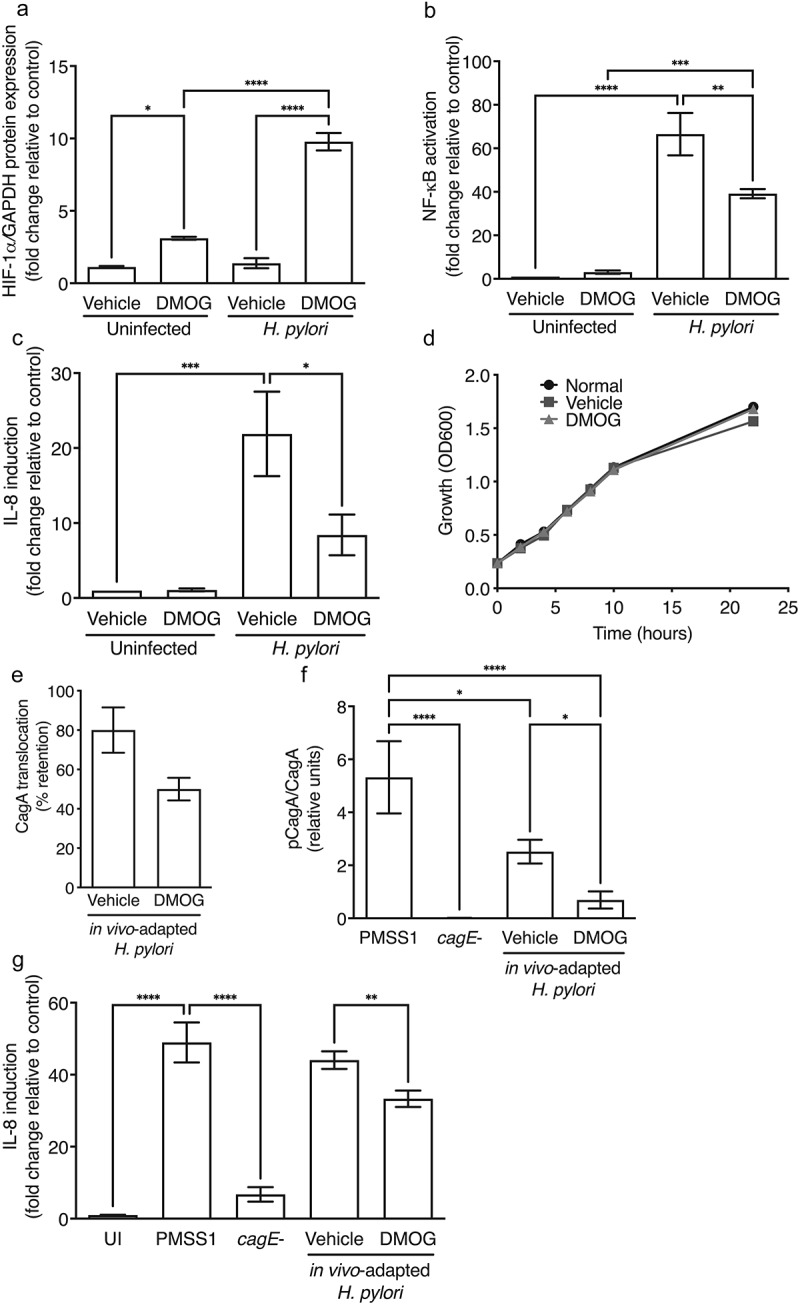
DMOG attenuates *H. pylori cag* T4SS function and induction of proinflammatory responses. Levels of HIF-1α were assessed by Western blot analyses (a), NF-κB activation by luciferase assay (b), and IL-8 by ELISA (c) in AGS cells treated with vehicle or DMOG and then challenged with or without *H. pylori*. *H. pylori* was grown in Brucella broth (BB) with vehicle or DMOG and bacterial growth was measured by OD600 (d). *In vivo*-adapted *H. pylori* were isolated from infected mice treated with vehicle or DMOG and co-cultured with AGS cells to assess CagA translocation by Western blot analysis (e-f) and IL-8 by ELISA (g). The percentage of *H. pylori* that retained T4SS function (e) and CagA translocation (f) were determined. ANOVAs with Sidak multiple comparisons test were used for statistical analyses and standard error of the mean is shown. ****, *P* < .0001; ***, *P* < .001; **, *P* < .01; *, *P* < .05.

To next assess direct effects of DMOG on *H. pylori* virulence, gastric epithelial cells were treated with vehicle or DMOG and then challenged with *H. pylori*. Concordant with the finding that HIF-1α is more robustly expressed in inflammatory immune infiltrates *in vivo* (Supplementary Figure 1A-C), HIF-1α expression was stabilized by DMOG in gastric epithelial cells *in vitro*, and was augmented with *H. pylori* infection (Figure 4a). However, consistent with the *in vivo* suppressive phenotype, DMOG significantly reduced *H. pylori*-induced NF-κB activation (Figure 4b) and IL-8 induction (Figure 4c). To confirm that DMOG did not exert adverse effects on *H. pylori* growth and viability, *H. pylori* were grown in the presence or absence of DMOG. There were no effects of DMOG on *H. pylori* growth or viability, compared to control (Figure 4d).

To increase the depth of our mechanistic analyses, we next assessed the effects of *in vivo*-DMOG-adaptation on the virulence potential of *H. pylori* isolates. *In vivo*-adapted *H. pylori* strains were isolated from mice treated with vehicle or DMOG, minimally passaged, and then co-cultured with gastric epithelial cells to assess translocation of the effector protein CagA and induction of the downstream proinflammatory chemokine IL-8. The input parental *H. pylori* strain PMSS1 and the PMSS1 *cagE*^−^ isogenic mutant were used as positive and negative controls, respectively, as the parental strain PMSS1 harbors the ability to translocate CagA and induce IL-8, while loss of *cagE* prevents CagA translocation and partially attenuates IL-8 induction. *In vivo*-adaptation to DMOG resulted in a marked reduction in the ability of output *H. pylori* strains to translocate CagA, whereby 50% of isolates exposed to DMOG completely lost the ability to translocate CagA, compared to only 20% of *H. pylori* isolates *in vivo*-adapted to vehicle (Figure 4e). Among strains that maintained the ability to translocate CagA, *in vivo*-adaptation to DMOG resulted in a significant reduction in levels of CagA translocation compared to isolates *in vivo*-adapted to vehicle (Figure 4f). Loss of CagA translocation was paralleled by significant reductions in IL-8 secretion (Figure 4g). These findings suggest that DMOG, while increasing the stabilization of HIF-1α, counterbalances this effect via a concomitant suppressive effect on *H. pylori cag* T4SS function and virulence, which may attenuate inflammation and facilitate persistence.

## Discussion

Hypoxia is a well-recognized condition affecting the development and progression of tumors via exerting an important role in therapeutic resistance, recurrence, and metastatic potential. HIF-1α, which is induced under hypoxic conditions, is an important transcriptional regulator of a number of signaling pathways that mediate the development and progression to cancer. In gastric cancer, HIF-1α has been shown to inhibit apoptosis, while promoting proliferation, angiogenesis, metastasis, and stemness of gastric cancer cells.^23^ Further, HIF-1α expression is associated with increased invasion and metastasis and reduced overall survival in gastric cancer patients.^12^ Recent studies have also described non-transcriptional functions for HIF-1α that contrast with its canonical roles in carcinogenesis, via contributions to cell cycle arrest^24,25^ and inhibition of DNA replication.^26^

Prolyl hydroxylases (PHDs) regulate the stability of hypoxia-inducible factors, and hydroxylase inhibitors, such as DMOG, result in the stabilization of HIF-1α. However, published data have identified an important paradox as DMOG also exerts a protective effect in certain models of inflammation. In the current study, we demonstrate that DMOG is protective against *H. pylori*-induced gastric inflammation and injury by attenuating host proinflammatory immune responses and M1 macrophage responses. Specifically, our findings demonstrate that DMOG decreases production of a subset of chemokines that are important in the recruitment and infiltration of innate immune components, particularly neutrophils. Further, DMOG significantly attenuates the induction of several potent Th1 proinflammatory cytokines, including IL-1β, IL-6, and TNF-α, all of which signal via type I cytokine receptors that are structurally divergent from other cytokine receptor types. In addition to dampening Th1 proinflammatory cytokine responses, DMOG also significantly attenuates the secreted M1 macrophage markers IFN-γ, IL-1β, IL-6, IL-12, and TNF-α, which in turn further dampens Th1 adaptive immune responses to *H. pylori* infection.

In addition to suppressive effects on the proinflammatory immune response, our findings also demonstrate that DMOG can attenuate *H. pylori* virulence mechanisms. However, although we have demonstrated that DMOG treatment significantly attenuates *cag* type IV secretion system function and concomitant CagA translocation, this study has not addressed the role of other important *H. pylori cag* T4SS effectors, adhesins, or other well characterized virulence factors.

Consistent with our results, DMOG is also protective in dextran-sodium sulfate (DSS)-induced colitis and attenuates production of the DSS-induced proinflammatory cytokines IL-1β, IL-6, IL-12, and TNF-α.^27^ In addition, concordant with our findings, several studies have demonstrated DMOG-mediated protection in a variety of models through alterations in macrophage polarization, with decreases in the M1 macrophage markers IL-1β, IL-6, and TNF-α and/or increases in the M2 macrophage markers IL-4, IL-10, Arg-1.^17–19^ In other models of microbial-induced injury, DMOG attenuates *Clostridium difficile* toxin-induced injury via decreasing proinflammatory chemokine and cytokine production,^28^ and significantly reduced mortality in a *Pseudomonas aeruginosa*-mediated model of pneumonia.^29^ DMOG protection has also been reported to be mediated through enhanced mucosal barrier function leading to decreased membrane permeability;^30–32^ we have not yet investigated the role of DMOG in enhanced barrier function in our model of *H. pylori-*induced gastric inflammation and injury, but our findings provide a framework for these studies in the future. Collectively, these studies demonstrate the importance of PHD inhibition in reducing the severity of disease in both chemically- and microbially-induced models of inflammation and injury through a variety of different mechanisms.

HIF-1α is induced by certain bacterial pathogens,^33,34^ while other bacterial pathogens are able to interfere with HIF-1α activation.^35,36^ Given the duality and complex role of HIF-1α in regulating inflammation, carcinogenesis, and immune-mediated bactericidal activity,^37^ the question of whether HIF-1α is beneficial or detrimental for *H. pylori*-induced disease is central. A prior study addressed this by demonstrating that myeloid HIF-1α contributes to proinflammatory responses induced by *H. pylori*, but is protective against *H. pylori*-mediated gastritis *in vivo*,^38^ emphasizing the complex interplay of the innate immune and inflammatory phenotypes in driving pathologic responses.

Collectively, these new data highlight the importance of HIF-1α in populations at high risk for gastric cancer, but also demonstrate that use of a PHD inhibitor, DMOG, and consequent stabilization of HIF-1α is protective in *H. pylori*-induced gastric inflammation and injury. Importantly, hydroxylase inhibitors, such as Roxadustat, have been clinically approved for other therapies^39^ and could potentially be repurposed. Thus, it is of paramount importance to more fully dissect the distinct cell-specific roles of HIF-1α in regulating host inflammatory responses as well as signaling pathways related to carcinogenesis as a means to identify actionable targets that can limit *H. pylori*-induced disease.

## Methods

### *Murine model of* H. pylori-*induced injury*

C57BL/6NHsd (Envigo #4405) mice and transgenic hypergastrinemic INS-GAS^+/+^ mice on a FVB/N background^40–42^ were bred and housed in the Vanderbilt University Medical Center animal care facilities. C57BL/6 mice were subjected to intraperitoneal injection of vehicle (PBS) or DMOG (8 mg, Cayman Chemicals #71210), every 48 hours for seven days prior to *H. pylori* challenge. Mice were orogastrically challenged with Brucella broth (BD Biosciences #211088) or with *H. pylori* strain PMSS1 and mice were euthanized eight weeks post-challenge, as previously described.^7^

### Histopathology

Gastric tissue was fixed, paraffin-embedded, and stained with hematoxylin and eosin (H&E). A pathologist assessed indices of gastric inflammation and injury as previously described.^7^ Severity of acute and chronic inflammation was graded 0–3 in both the gastric antrum and corpus for a cumulative score of 0–12.

### Chemokine and cytokine multiplex bead array

Gastric tissue was lysed (CelLytic MT Cell Lysis Reagent, Sigma #C3228) and lysates were diluted 1:3 and mixed with magnetic beads according to the manufacturer’s instructions (MILLIPLEX Cytokine/Chemokine Magnetic Bead Panel, Millipore MCYTOMAG-70K-PMX) and as previously described.^7^ Data were analyzed via Millipore software platform and standardized to protein concentrations.

### Human gastric epithelial cell culture

AGS human gastric epithelial cells (ATCC CRL-1739, mycoplasma-negative) were grown in RPMI 1640 with L-glutamine (Corning #10–040-CV) supplemented with 10% fetal bovine serum (FBS, Atlanta Biologicals #S11150), and HEPES buffer (1 mM, Corning #25–060-Cl) at 37°C with 5% CO_2_, as previously described.^43^ AGS cells were co-cultured with *H. pylori* at a multiplicity of infection (MOI) of 100:1 for six hours unless otherwise noted.

### Western blot analysis

Protein lysates from AGS:*H. pylori* co-cultures were separated by SDS-PAGE and transferred to PVDF membranes (Thermo Scientific #88518). Levels of total CagA (anti-CagA antibody, Austral Biologicals #HPP-5003-9) and phosphorylated CagA (anti-pY99 antibody, Santa Cruz #sc-7020) were determined and quantified using ImageJ 1.50i, as previously described.^7^

### NF-κB activation assay

*H. pylori* were co-cultured with NF-κB luciferase gastric epithelial (AGS) reporter cells at a MOI of 10:1 for four hours, and NF-κB activation was measured using a Steady-Glo luciferase assay substrate according to the manufacturer’s instructions (Promega #E2510), as previously described.^6^

### IL-8 ELISA

Levels of IL-8 were quantified in AGS:*H. pylori* co-culture supernatants by Quantikine® IL-8 ELISA (R&D Systems #D8000C) according to the manufacturer’s instructions. Data were analyzed with Gen5 software (Synergy4, BioTek), as previously described.^7^

### Statistical analysis

Mean values are shown with standard error of the mean (SEM) for experiments performed and replicated on at least three independent occasions. Unpaired parametric t-tests and one-way ordinary ANOVAs with Sidak multiple comparison test were used for statistical analyses. When data did not assume normal Gaussian distribution, either a nonparametric Mann Whitney t test or a Kruskal-Wallis ANOVA test with Dunn’s multiple comparisons test were used. All analyses were performed using GraphPad Prism. *P* < .05 was considered statistically significant (****, *P* < .0001; ***, *P* < .001; **, *P* < .01; *, *P* < .05).

### Ethics

All animal and human studies were conducted in accordance with the Declaration of Helsinki principles and have been approved by the VUMC Institutional Animal Care and Use Committee (IACUC) and the Institutional Review Board (IRB), respectively. The IRB of Louisiana State University Health Sciences Center, the Institutional Review Committee of Memorial Medical Center in New Orleans, Louisiana, the Committees on Ethics of Universidad del Valle and Hospital Departamental de Nariño in Colombia, the Committees on Ethics of Pontificia Universidad Catolica de Chile, and the IRB of Vanderbilt University Medical Center approved these protocols.

## Supplementary Material

Supplemental Material

## Data Availability

The data that support the findings of this study are available in figshare at https://doi.org/10.6084/m9.figshare.22154753.v2, Noto, Jennifer (2023): MILLIPLEX Cytokine/Chemokine Magnetic Bead Panel. figshare. Dataset. https://doi.org/10.6084/m9.figshare.22154753.v2.
