# Supplementary material for: Targeting hypoxia-inducible factor-1 alpha suppresses Helicobacter pylori-induced gastric injury via attenuation of both cag-mediated microbial virulence and proinflammatory host responses
Source: Gut Microbes. 2023 Oct 13;15(2):2263936. doi: 10.1080/19490976.2023.2263936 (PMC10578190; doi:10.1080/19490976.2023.2263936)
Supplement: Supplemental Material [file KGMI_A_2263936_SM8643.zip › KGMI_supplemental material/3_Noto_Gut Microbes_Brief Report_Supplemental_revised resubmission_no underline.docx]

**Supplementary Methods**

***Human gastric tissue samples.*** The Institutional Review Board (IRB) of Louisiana State University Health Sciences Center, the Institutional Review Committee of Memorial Medical Center in New Orleans, Louisiana, the Committees on Ethics of Universidad del Valle and Hospital Departamental de Nariño in Colombia, the Committees on Ethics of Pontificia Universidad Catolica de Chile, and the IRB of Vanderbilt University Medical Center approved these protocols. Gastric tissues used for immunohistochemistry (IHC) were selected from a previous study involving patients attending gastrointestinal clinics in New Orleans, Louisiana^1, 2^ and from archival material derived from gastrectomy specimens from patients with gastric cancer in Santiago, Chile.^3^ Demographics and histopathological diagnoses of individuals included in the IHC analysis are included in Supplementary Table 1.

Patients from New Orleans (with available gastric biopsy tissues for IHC) were randomly selected by diagnostic group from a large cohort recruited between 1995 and 2005.^2^ Briefly, adults undergoing upper GI endoscopy at the Medical Center of Louisiana and the Oschner Baptist Medical Center (formerly Memorial Medical Center) were invited to participate. Although race was not a factor considered for the random selection of samples, the majority of cases with normal gastric mucosa were non-Hispanic White. In contrast, among the 25 individuals with gastritis or more advanced gastric histology, the majority were African American (Supplementary Table 1). In the United States, gastric cancer incidence and mortality rates are approximately two times higher in non-Hispanic African Americans than in non-Hispanic Whites,^4^ which places this specific population at higher risk for gastric cancer.

Gastric biopsies used for *H. pylori* cultures were collected from patients from low gastric cancer risk (Pacific coastal town of Tumaco) and high gastric cancer risk (Andean mountain town of Túquerres) regions of Colombia.^5^ Demographics and histopathological diagnoses of patients from which *H. pylori* clinical isolates were obtained as well as *H. pylori* genotypes and MLST classifications are included in Supplementary Table 2.

***HIF-1α and CA9 immunohistochemistry.*** HIF-1α immunohistochemistry (IHC) analysis was performed on human gastric tissue samples and murine gastric tissue sections (C57BL/6 and transgenic hypergastrinemic INS-GAS^+/+^ mice on a FVB/N background) using a rabbit polyclonal anti-HIF-1α antibody (Novus Biologicals #NB100-134) and scored by a single pathologist blinded to treatment groups, as previously described.^6^ The percentage of positive cells was assessed in a semiquantitative manner to determine the approximate proportion of epithelial cells that were positively stained for HIF-1α. The intensity of epithelial staining was also graded on a scale of 0-3 (absent (0), weak (1), moderate (2), or strong (3)). To calculate the IHC score, the percentage of epithelium stained was multiplied by the corresponding intensity score (0-3) for a total maximum score of 300. In tissues from gastric cancer patients, staining was scored separately in epithelium with low-grade dysplasia or intestinal metaplasia, whenever possible. In mice, in addition to quantifying HIF-1α-positive cells in the epithelium, HIF-1α-positive cells were also specifically enumerated in polymorphonuclear neutrophils (PMNs) and mononuclear leukocytes from five high-powered fields (HPF, 400X); the average number of HIF-1α-positive cells/HPF are shown.

Carbonic anhydrase IX (CA9) IHC was performed on C57BL/6 murine gastric tissue sections using a rabbit polyclonal anti-CA9 antibody (Invitrogen #PA1-16592) and scored by a single pathologist blinded to treatment groups. Staining was predominantly localized to epithelial cells within the basolateral membrane of the gastric corpus, and quantification of CA9 staining represents the portion of the oxyntic mucosa that exhibited strong cytoplasmic staining in epithelial cells and predominantly localized to chief cells.

***HIF-1α* qRT-PCR*.*** *H. pylori* clinical isolates were co-cultured with AGS cells at a MOI of 100:1 for six hours and RNA was extracted. qRT-PCR was performed to quantify levels of *HIF-1α* (ThermoFischer, Applied Biosystems #Hs00153153_m1) normalized to levels of *GAPDH* (ThermoFischer, Applied Biosystems #4310884E) according to the manufacturer’s instructions.

***H. pylori strains and growth conditions.*** Wild-type *H. pylori* *cag^+^* strain PMSS1 or its PMSS1 *cagE^-^* (*cag* secretion system ATPase) isogenic mutant (as a negative control for experiments) were cultured on trypticase soy agar with 5% sheep blood agar plates (Remel #01198) for *in vitro* passage. Isogenic mutants were also cultured on Brucella agar (BD Biosciences #211086) plates containing 20 μg/ml kanamycin (Sigma #K-4000) to confirm presence of the kanamycin antibiotic resistance cassette. *H. pylori* clinical strains were previously isolated from patients from low gastric cancer risk (Pacific coastal town of Tumaco) and high gastric cancer risk (Andean mountain town of Túquerres) regions of Colombia (Supplementary Table 2).^5^ *H. pylori* strains were then cultured in Brucella broth (BB, BD Biosciences #211088) supplemented with 10% fetal bovine serum (FBS, Atlanta Biologicals #S11150) for 16 to 18 hours at 37°C with 5% CO_2_ as previously described.^6, 7^

***H. pylori biochemical testing and genotyping***. *H. pylori* culture was performed from one antral biopsy. *H. pylori* urease, catalase, and oxidase (BD Biosciences #231746) testing were determined using specified biochemical tests. *H. pylori cagA* and *vacA* genotypes and MLST classifications were previously determined.^5^ *H. pylori sabA* and *babA* genotypes were determined by PCR amplification using the following primers for *sabA* (F: ACTAGGCGATTCCTCCAAAAAGCC and R: CTGACGCAAAACCTAGCCAACACC) and *babA* (F: ACTACGGCTTTTACATGAGTGCGG and R: AGTAGGCGAAAGCAAAAGATGGG) using both *H. pylori* PMSS1 and 7.13 strains as templates. Data undetermined (UN) indicates that no PCR band was detected for *babA*, but does not indicate the absence of the *babA* gene, as there could be sequence variations in the clinical strains.

***H. pylori quantitative culture.*** Gastric tissue was harvested from mice and homogenized in sterile phosphate-buffered saline (PBS). Following serial dilution, samples were plated on selective trypticase soy agar plates with 5% sheep blood (Remel #R01198) containing vancomycin (20 μg/ml, Sigma-Aldrich #V-2002), nalidixic acid (10 μg/ml, Sigma-Aldrich #N-4382), bacitracin (30 μg/ml, Millipore #1951), and amphotericin B (2 μg/ml, Sigma-Aldrich #195043) for isolation of *H. pylori*. Plates were incubated for five days at 37°C with 5% CO_2_ and colonies were identified as *H. pylori* based on characteristic morphology, Gram stain (BD Biosciences #212539), urease, catalase, and oxidase (BD Biosciences #231746) activities, as previously described.^6^ Colonization efficiency is defined as the number of mice successfully colonized and is represented as a percentage. Colonization density is expressed as log colony-forming units/gram of tissue. *In vivo*-adapted single-colony *H. pylori* isolates were harvested from infected mice treated with vehicle or DMOG for subsequent analyses.

***Primary ex vivo gastric epithelial organoid and immune cell culture.*** Mouse primary gastric epithelial cell monolayers were generated as reported previously.^8^ Briefly, gastric glands harvested from uninfected transgenic hypergastrinemic INS-GAS^+/+^ mice on an FVB/N background were embedded into Matrigel (Corning #356231) and cultured in 50% L-WRN conditioned media at 37°C and 5% CO_2_. Once glands formed 3D gastric organoids, they were trypsinized and plated on collagen-coated plates or transwell filters (Costar #3460) in 5% L-WRN conditioned media at 37°C and 5% CO_2_ to convert to 2D monolayers.

Autologous bone marrow-derived macrophages were obtained from femurs of uninfected INS-GAS mice, as previously described.^8^ Briefly, bone marrows were treated with ACK red blood cell lysis buffer (Becton Dickinson #RGC-3015) and washed with PBS, and recovered monocytes were plated in DMEM media (Gibco #12430-054) supplemented with 10% FBS (R&D Systems #S11150), 1% HEPES (Corning #25-060-Cl), 1% penicillin-streptomycin (Gibco #15140122), and 20 ng/mL of M-CSF (PeproTech #315-02) for seven days at 37°C and 5% CO_2_ for differentiation.

Autologous splenocytes were isolated from the spleens of uninfected INS-GAS mice. Splenocytes were recovered in in RPMI 1640 (Gibco #22400-089) with 10% FBS (R&D Systems #S11150), and 1% penicillin-streptomycin (Gibco #15140122) and supplemented with 0.2 μg/mL anti-CD3 (R&D Systems #MAB100R) and 0.1 μg/mL anti-CD28 (R&D Systems #MAB4832) for one hour to induce T cell activation.

Gastric epithelial monolayers were seeded in the upper chamber of a transwell system (Costar #3460) to form 2D monolayers, while 2x10⁶ autologous bone marrow-derived macrophages and 1x10⁶ splenocytes, treated with anti-CD3 and anti-CD28, were placed in the lower chamber. Epithelial gastric organoid cell monolayers were then infected with *H. pylori* (MOI 10:1) and chemokine and cytokine expression was quantified by qRT-PCR from macrophage/T cell lysates, as previously described.^8-10^

Supplementary References:

1. Zabaleta J, Camargo MC, Piazuelo MB, Fontham E, Schneider BG, Sicinschi LA, Ferrante W, Balart L, Correa P, Ochoa AC. Association of interleukin-1beta gene polymorphisms with precancerous gastric lesions in African Americans and Caucasians. Am J Gastroenterol 2006; 101:163-171.

2. Zabaleta J, Camargo MC, Ritchie MD, Piazuelo MB, Sierra RA, Turner SD, Delgado A, Fontham ET, Schneider BG, Correa P, et al. Association of haplotypes of inflammation-related genes with gastric preneoplastic lesions in African Americans and Caucasians. Int J Cancer 2011; 128:668-675.

3. Araya JC, Anabalon L, Roa I, Bravo M, Villaseca MA, Guzman P, Roa JC. Association between *Helicobacter pylori* genotype and the severity of gastritis in infected adults. Rev Med Chil 2004; 132:1345-1354.

4. Giaquinto AN, Miller KD, Tossas KY, Winn RA, Jemal A, Siegel RL. Cancer statistics for African American/Black People 2022. CA Cancer J Clin 2022; 72:202-229.

5. de Sablet T, Piazuelo MB, Shaffer CL, Schneider BG, Asim M, Chaturvedi R, Bravo LE, Sicinschi LA, Delgado AG, Mera RM, et al. Phylogeographic origin of *Helicobacter pylori* is a determinant of gastric cancer risk. Gut 2011; 60:1189-1195.

6. Noto JM, Piazuelo MB, Shah SC, Romero-Gallo J, Hart JL, Di C, Carmichael JD, Delgado AG, Halvorson AE, Greevy RA, et al. Iron deficiency linked to altered bile acid metabolism promotes *Helicobacter pylori*-induced inflammation-driven gastric carcinogenesis. J Clin Invest 2022; 132:e147822.

7. Noto JM, Rose KL, Hachey AJ, Delgado AG, Romero-Gallo J, Wroblewski LE, Schneider BG, Shah SC, Cover TL, Wilson KT, et al. Carcinogenic *Helicobacter pylori* strains selectively dysregulate the *in vivo* gastric proteome, which may be associated with stomach cancer progression. Mol Cell Proteomics 2019; 18:352-371.

8. Suarez G, Romero-Gallo J, Piazuelo MB, Sierra JC, Delgado AG, Washington MK, Shah SC, Wilson KT, Peek RM, Jr. Nod1 Imprints inflammatory and carcinogenic responses toward the gastric pathogen *Helicobacter pylori*. Cancer Res 2019; 79:1600-1611.

9. Latour YL, Sierra JC, Finley JL, Asim M, Barry DP, Allaman MM, Smith TM, McNamara KM, Luis PB, Schneider C, et al. Cystathionine gamma-lyase exacerbates *Helicobacter pylori* immunopathogenesis by promoting macrophage metabolic remodeling and activation. JCI Insight 2022; 7:e155338.

10. Latour YL, Sierra JC, McNamara KM, Smith TM, Luis PB, Schneider C, Delgado AG, Barry DP, Allaman MM, Calcutt MW, et al. Ornithine decarboxylase in gastric epithelial cells promotes the immunopathogenesis of *Helicobacter pylori* infection. J Immunol 2022; 209:796-805.

Supplementary Figure Legends:

Supplementary Figure 1. *H. pylori* and DMOG increases HIF-1α expression in C57BL/6 mice *in vivo*. The average number of HIF-1α^+^ cells/HPF was assessed in uninfected (UI, N=6) or *H. pylori*-infected mice (N=10) (A-C). The number HIF-1α^+^ cells/HPF was assessed in *H. pylori*-infected mice treated with either vehicle (N=10) or DMOG (N=12) and standardized to the level of total inflammation in each group (D). CA9 staining was quantified as a percentage of the oxyntic mucosa that exhibited strong cytoplasmic staining in epithelial cells and predominantly localized to chief cells (E). Representative IHC images are shown at 400X and scale bars represent 50 μm (B-C). Arrows designate regions of positive staining in immune cells. Open symbols: uninfected mice; closed symbols: *H. pylori*-infected mice. Circles: vehicle-treated; squares: DMOG-treated. Each point represents data from an individual mouse. Unpaired parametric t-tests were used for statistical analyses and standard error of the mean is shown. ***, P<0.001; **, P<0.01; *, P<0.05

Supplementary Figure 2. Chemokines and cytokines not significantly altered by *H. pylori* infection or DMOG treatment. Chemokines and cytokines were analyzed in gastric tissue from mice treated with vehicle or DMOG and then challenged with or without *H. pylori*. Chemokines MCP-1 (A), MIP-1α (B), and MIP-2 (C) as well as proinflammatory cytokines GM-CSF (D), IL-1α (E), IL-2 (F), IL-5 (G), IL-9 (H), and IL-15 (I), and anti-inflammatory cytokines IL-4 (J), IL-10 (K), and IL-13 (L) were not significantly altered with DMOG treatment. Open symbols: uninfected mice; closed symbols: *H. pylori*-infected mice. Circles: vehicle-treated; squares: DMOG-treated. ANOVAs with Sidak multiple comparisons test were used for statistical analyses.

Supplementary Figure 3. *H. pylori* induces proinflammatory chemokines and cytokines using enriched primary *ex vivo* model system derived from a mouse model prone to gastric carcinogenesis. The average number of HIF-1α^+^ cells/HPF was assessed in uninfected (UI, N=6) or *H. pylori*-infected mice (Hp, N=10) (A-C). Representative IHC images are shown at 400X and scale bars represent 50 μm (B-C). Arrows designate regions of positive staining in immune cells. Gastric organoids were isolated from uninfected INS-GAS mice and cultured with autologous macrophages and T cells in a transwell system. Epithelial gastric organoid monolayers were then infected *ex vivo* with *H. pylori* and chemokine and cytokine expression was quantified from macrophage/T cell lysates by qRT-PCR (D-J). *H. pylori*-induced increased levels of the chemokines *Kc* (D) and *Rantes* (F) as well as levels of the proinflammatory cytokines *Il1β* (F), *Il17* (G), *Tnfα* (H), *Il9* (I), and *Il22* (J). Open circles: uninfected; closed circles: *H. pylori*-infected. Unpaired parametric t-tests were used for statistical analyses and standard error of the mean is shown. ***, P<0.001; **, P<0.01; *, P<0.05
