# Supplementary figures and images for: Targeting hypoxia-inducible factor-1 alpha suppresses Helicobacter pylori-induced gastric injury via attenuation of both cag-mediated microbial virulence and proinflammatory host responses
Source: Gut Microbes. 2023 Oct 13;15(2):2263936. doi: 10.1080/19490976.2023.2263936 (PMC10578190; doi:10.1080/19490976.2023.2263936)

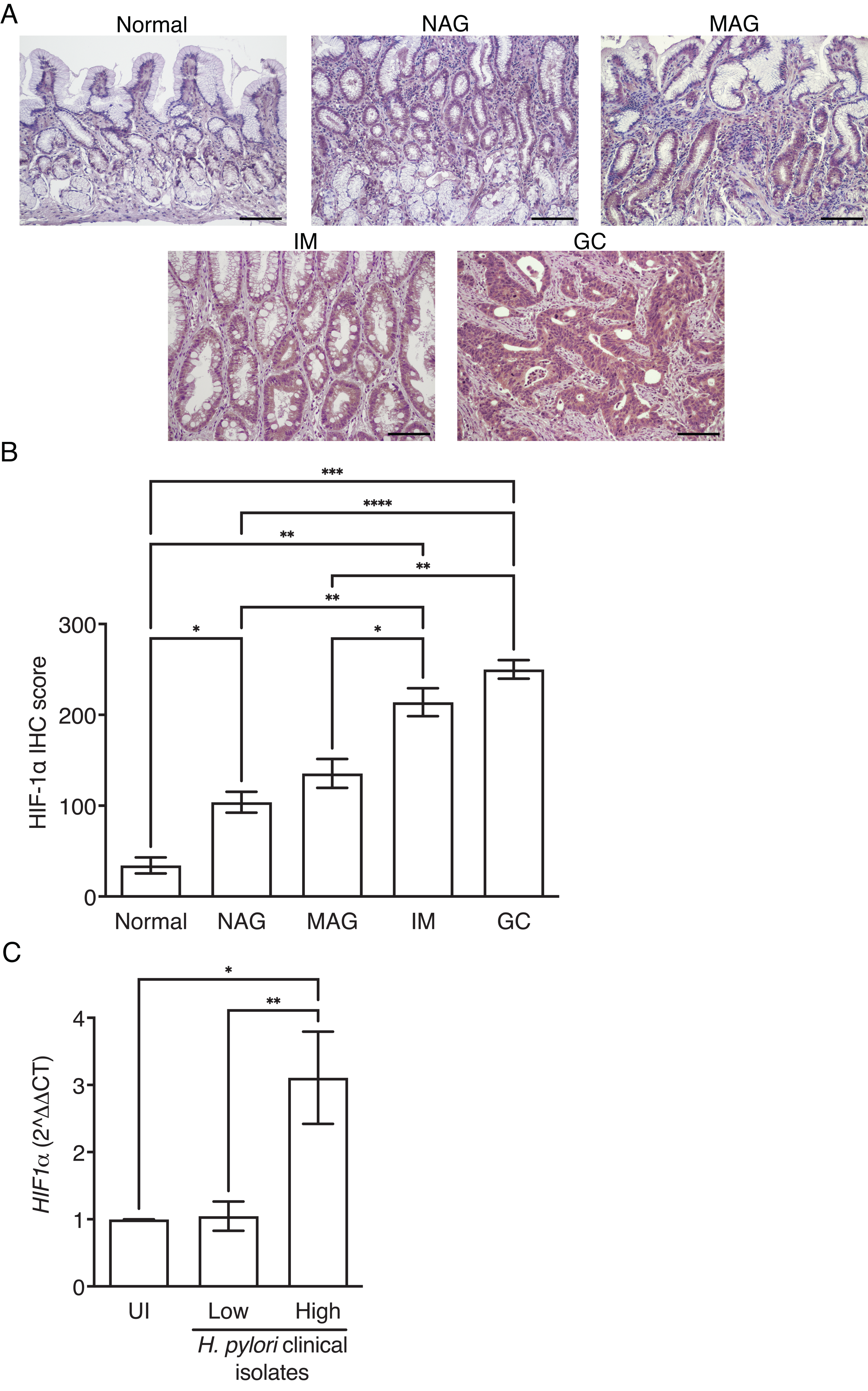

Supplement: Supplemental Material [file KGMI_A_2263936_SM8643.zip › KGMI_supplemental material/Supplementary Figure 1_resubmission.tif]

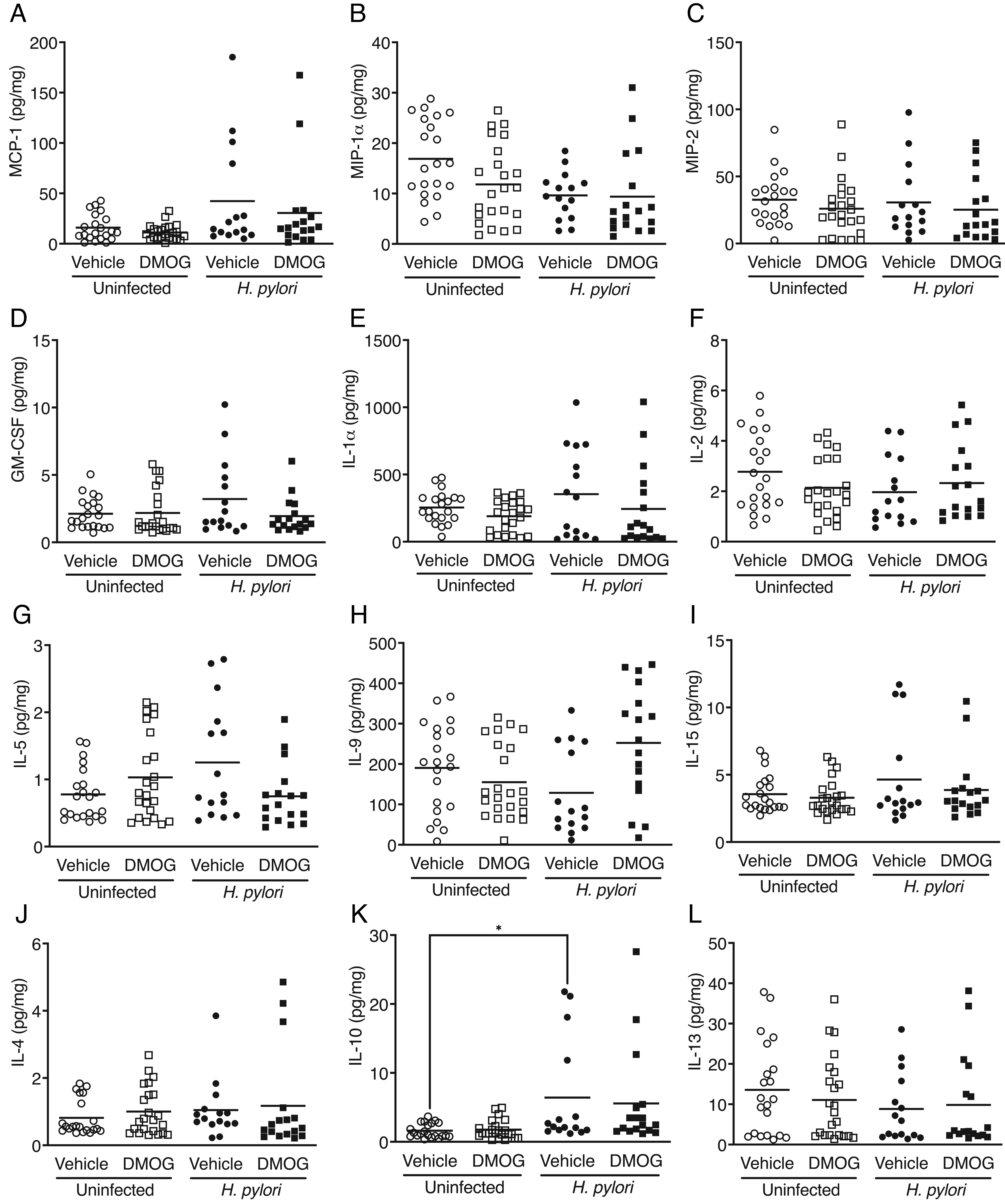

Supplement: Supplemental Material [file KGMI_A_2263936_SM8643.zip › KGMI_supplemental material/Supplementary Figure 2_resubmission.tif]

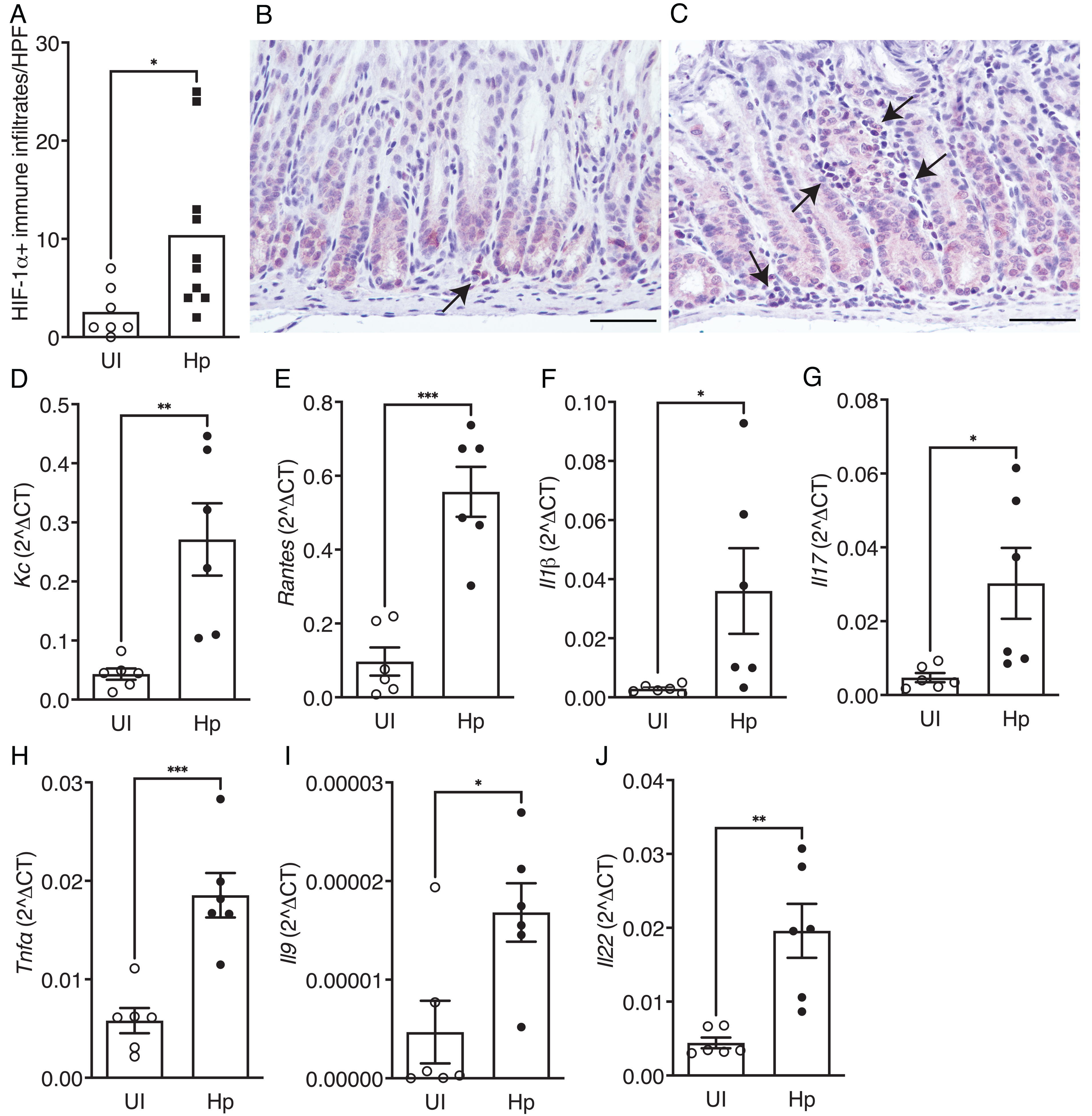

Supplement: Supplemental Material [file KGMI_A_2263936_SM8643.zip › KGMI_supplemental material/Supplementary Figure 3_resubmission.tif]
